# Agassiz—Comments of the French Press on His Work in This Country

**Published:** 1874-02-15

**Authors:** 


AGASSIZ. — COMMENTS OF THE FRENCH PRESS ON HIS WORK IN THIS COUNTRY.—Who will say, now, that "they do these things better in France?" Let the French themselves respond. *La France Medicale* of December 24, 1873, in an obituary record of the distinguished naturalist Agassiz, comments as follows upon his residence in this country:

"He found, among the Americans, co-operators who were ready to place at his disposal resources of every kind, infinitely superior to those which European States in general, and France in particular, allot to scientific research and instruction. Thus, on one occasion, being solicited in the public journals to undertake an investigation of the fishes of foreign seas, the voice of the press was so potent in its appeal that gifts poured in from every quarter, and at the end of a few years the collection at New Cambridge became the richest in the world. On another occasion, a special society was organized for the exclusive object of furthering an exploration of the rivers and coasts of Brazil, by the same naturalist, in the interests of ichthyology. Latterly, also, a wealthy American gentleman placed at his disposal an entire island, for the purpose of its conversion into a zoological garden. (!) In 1859, the French Government offered for his acceptance the chair of Paleontology at the Museum, vacated by the death of Dorbigny, but it was refused. No greater success attended similar efforts made in 1867. How, the deuce, could we expect that Agassiz would come to our poor France, where the entire amount of perquisites allowed for instruction of the most valuable character would not equal even the sums placed at his service by individual Americans, to enable him to prosecute his scientific researches?"

